# A Pilot Study to Assess Inhalation Exposures among Sugarcane Workers in Guatemala: Implications for Chronic Kidney Disease of Unknown Origin

**DOI:** 10.3390/ijerph17165708

**Published:** 2020-08-07

**Authors:** Joshua W. Schaeffer, John L. Adgate, Stephen J. Reynolds, Jaime Butler-Dawson, Lyndsay Krisher, Miranda Dally, Richard J. Johnson, Katherine A. James, Diana Jaramillo, Lee S. Newman

**Affiliations:** 1 Department of Environmental and Occupational Health, Colorado School of Public Health, University of Colorado, Aurora, CO 80045, USA; John.Adgate@ucdenver.edu (J.L.A.); stephen.reynolds@colostate.edu (S.J.R.); jaime.butler-dawson@cuanschutz.edu (J.B.-D.); lyndsay.krisher@cuanschutz.edu (L.K.); miranda.dally@cuanschutz.edu (M.D.); kathy.james@cuanschutz.edu (K.A.J.); lee.newman@cuanschutz.edu (L.S.N.); 2 Department of Environmental and Radiological Health Sciences, Colorado State University, Fort Collins, CO 80523, USA; 3 Center for Health, Work and Environment, Colorado School of Public Health, University of Colorado, Aurora, CO 80045, USA; DIANA.JARAMILLO@UCDENVER.EDU; 4 Division of Renal Diseases and Hypertension, Department of Medicine, School of Medicine, University of Colorado, Aurora, CO 80045, USA; RICHARD.JOHNSON@CUANSCHUTZ.EDU; 5 Department of Epidemiology, Colorado School of Public Health, University of Colorado, Aurora, CO 80045, USA; 6 Division of Pulmonary Sciences and Critical Care Medicine, Department of Medicine, School of Medicine, University of Colorado, Aurora, CO 80045, USA

**Keywords:** particulate matter, exposures, kidney, CKDu, sugarcane workers, silica

## Abstract

Background: Sugarcane workers in Central America experience a heavy burden of chronic kidney disease of unknown origin. We conducted a pilot study among worker proxies in Guatemala to characterize exposures to particulate matter, silica, heavy metals, and glyphosate, as well as to examine potential nephrotoxic exposures. Methods: Air, soil, and ash samples were collected and analyzed using scanning electron microscopy, X-ray diffraction, inductively coupled plasma mass spectrometry, and an enzyme-linked immunosorbent assay. Results: The average mass concentration for particulate matter (PM)_2.5_ and PM_100_ exposures were 360 µg/m^3^ (range: 32 to 1500 µg/m^3^) and 555 µg/m^3^ (range: 229 to 1170 µg/m^3^), respectively. The elemental composition of particles was largely silicon. The amount of crystalline silica was below 5 μg, yet the percentage of total silica was ~17% by weight. Putatively, the silica was in the amorphous form. Concentrations of aluminum and calcium ranged from 2–7 μg/m^3^. Glyphosate was not detectable in analyzed air samples but was detectable at concentrations ranging from 81–165 ppb in soil samples. Conclusion: Sugarcane workers are exposed to high concentrations of particulate matter. Future studies should investigate the potential role of silica, heavy metals, and agrochemicals in the etiology of chronic kidney disease in this population.

## 1. Introduction

Agricultural workers across the world experience a disproportionate burden of chronic kidney disease of unknown origin (CKDu) [1,2,3,4,5,6,7,8,9]. The etiology remains uncertain; the disease is not related to traditional risk factors such as diabetes, hypertension, aging, or glomerular disease [9,10,11,12,13]. In Central America, CKDu (also referred to as Mesoamerican nephropathy) has been predominantly identified among sugarcane workers and is estimated to have caused over 20,000 deaths in the last 10 years [11]. It principally affects men aged between their 20s and 40s, and results in chronic tubulointerstitial nephritis [14]. Studies suggest that recurrent dehydration, exposure to high temperatures, and physically demanding working conditions contribute to CKDu risk [15,16,17,18,19]. Proposed contributing environmental exposures that have not been thoroughly examined include air pollutants, heavy metals, and agrochemicals [1]. There is an urgent need to address the causes and propose improvements in workplace and environmental conditions to benefit this vulnerable working population.

There are limited data related to particulate matter (PM) exposure and attendant health impacts among sugarcane workers [20,21,22,23,24,25]. To date, the existing studies assessing the impacts of PM exposure have focused on respiratory impacts and cardiovascular disease [21,22,23,24,25,26]. However, little is known about the deleterious effects of PM exposure on the kidney. A few recent studies in United States veteran and Medicare populations have found that ambient PM with a ≤2.5 µm (PM_2.5_) diameter concentration is associated with a higher risk of declining kidney function, incident CKD, and end stage renal disease [27,28,29]. Although mechanisms underlying the relationship of PM exposure and kidney dysfunction are not clear, it is known that particles less than 10 microns and, in particular ultrafine particles [30], can penetrate the pulmonary region of the lungs and enter the circulatory system. These particles can pass through the glomeruli into the urine with reabsorption into renal tubular cells [31]. Due to their high metabolic function, the tubules are especially sensitive to PM_2.5_, heavy metals, and other toxicants [31]. 

There is a need to identify and characterize potentially preventable nephrotoxic exposures that may put workers at risk for the development of CKDu [1]. Recent research suggests that occupational exposure to respirable silica increases the risk for traditional CKD [32,33,34,35] and targets proximal tubular cells [36]. Silica is an abundant mineral that may exist in different forms in soil and can accumulate in sugarcane plants [37]. Manual harvesting of sugarcane is preceded by burning the fields to remove dead foliage and facilitate cutting. The burning process contributes to local air pollution and produces a thick layer of ash on the ground at the base of the plants. This mixture of soil and ash is continuously aerosolized by the workers during cutting and stacking activities; silica may be present on these aerosolized particles [37,38,39]. Beyond the potential for adverse respiratory health outcomes, sugarcane workers are potentially at risk for silica-induced kidney injury. While silica is often cited as a potential risk factor for CKDu, very few studies have been conducted that evaluate it as an inhalation hazard.

In addition to silica, sugarcane workers may be exposed to other nephrotoxicants and contaminants. Guatemala has 37 volcanoes, four of which are active and in close proximity to the sugarcane growing region. As such, the top soil in the sugarcane fields may contain heavy metals, such as arsenic, cadmium, and nickel from the deposition of volcanic ash [40]. Exposure to these heavy metals and others are widely recognized as risk factors for kidney injury and disease [41,42]. Another potentially nephrotoxic chemical of concern is glyphosate [43], which is a non-specific herbicide that is applied to sugarcane to increase crop yields [44]. While precipitation decreases the amount of glyphosate in ambient air, the sugarcane harvest in Guatemala takes place during the dry season. Therefore, the accumulation of glyphosate, heavy metals, and silica in the soil could result in airborne exposure, especially during the dry season.

This manuscript describes the findings of a pilot study designed to characterize levels of PM, silica, heavy metals, and glyphosate while sugarcane fields were being burned and harvested. In this study, we collected air samples from the breathing zone of worker proxies, as well as from fixed locations in the sugarcane field. We also report levels of these compounds in settled ash and soil collected concurrently during air sampling.

## 2. Materials and Methods

This study was conducted in the fields adjacent to a large sugarcane mill in southwest Guatemala in April 2018. At this mill, the majority of the sugarcane is harvested manually by workers using a machete to cut at the base of the plant. Workers spend approximately 9–10 h in the field per day, six days per week, cutting, trimming, stacking cane, and taking rest breaks, as well as one lunch break. Pre-harvest burns are conducted a few hours prior to workers entering the field to remove leaves and other vegetation, as well as to increase sugar yield. During these pre-harvest burns, workers are cutting and stacking sugarcane in neighboring fields. Workers wear goggles and protective clothing (i.e., hat, long-sleeved shirt, gloves, wrist/shin guards, and boots); no respiratory protection is used. Work setting and work practice details have been previously described [45,46]. 

**Figure 1:**
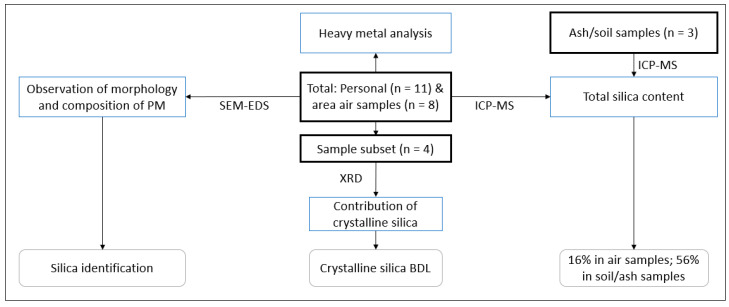
Overview of sample analysis plan used to characterize the presence and concentration of particulate matter and nephrotoxic contaminants during burning and harvesting of sugarcane. Abbreviations: SEM-EDS: scanning electron microscopy-energy dispersive spectroscopy; XRD: X-ray diffraction; XPS: X-ray photoelectron spectroscopy; ICP-MS: induced coupled plasma mass spectrometry; PM: particulate matter; BDL: below detection limit.

Two single day air sampling campaigns (here called Sampling Campaign 1 and 2) were conducted in two different fields with one intervening day. Sample collection during Campaign 1 took place one day after pre-harvest burning of sugarcane fields. Personal air samples were collected on worker proxies, i.e., study investigators in the field who also performed worker tasks while area-based samples were collected alongside manual cane cutting activities by company workers. Nearby fields were also being burned to prepare them for subsequent harvest. Worker proxies were typically 100 m or more away from smoke plumes generated during the sampling campaign. Sampling Campaign 2 was scheduled to collect personal and area-based samples during a pre-harvest burn while manual cane cutting activities occurred. Additionally, soil and ash samples were collected in conjunction with the active air sampling during and after pre-harvest burns in Sampling Campaigns 1 and 2. Collectively, these samples were analyzed for target nephrotoxic compounds using the techniques summarized in Figure 1. 

### 2.1. Sample Collection

#### 2.1.1. Particulate Matter Sample Collection

Two different size-fractions of PM were collected during both sampling campaigns using personal aerosol samplers and fixed monitoring stations that collected area samples at the approximate center of each field. Those particles with an aerodynamic diameter of ≤2.5 micrometer (μm; PM_2.5_) were collected using a BGI Cyclone sampler (Mesa Labs, Butler, NJ, USA) while PM with an aerodynamic diameter of ≤100 μm (PM_100_) was collected using Button samplers (SKC Inc., Eighty-Four, PA, USA). Area samples were collected using co-located Cyclone and Button samplers that were mounted at approximately 1.5 m (m) above ground level.

Ethics review and approval for the study was received from the Colorado Multiple Institutional Review Board (COMIRB #17–1328) and ZUGUEME Comité Ética Independiente in Guatemala. Accordingly, three members of the research team were outfitted with side-by-side aerosol samplers to collect PM_2.5_ and PM_100_ during a typical work shift. Each proxy worker wore two sampling pumps (one attached to the BGI Cyclone sampler and the other to the SKC Button sampler) to actively draw air into each sampler at 4 L per minute (L/min). The cyclone and Button samplers were loaded with 37-mm (mm) and 25 mm polyvinyl chloride filter (PVC; SKC Inc., Eighty-Four, PA, USA), respectively, with a pore size of 5.0 μm. All study pumps were calibrated to 4 L/min before and after sampling using a primary flow standard.

All air filters were securely transported back to Colorado State University and subsequently desiccated for 24 h and static neutralized using a U-Electrode (Mettler-Toledo, Inc.) before each pre- and post–sampling gravimetry. The total mass of each PM size fraction collected was determined by weighing a filter using a Mettler MT5 balance (Mettler-Toledo, Inc., Columbus, OH, USA). Time-weighted averages were determined by dividing the weight of PM on each filter by the volume of air sampled ranging from approximately 0.25 to 1.9 m^3^. Laboratory and field blank filters were used to correct for measurement error and background.

#### 2.1.2. Settled Ash and Soil Collection

Settled ash and soil samples were collected from the sugarcane fields. A settled ash catchment system (a 2 m^2^ ground tarp) was deployed in proximity to the fixed monitoring stations during the sampling campaign. Settled ash was collected by sweeping with pre-cleaned hand brush and dustpan and then transferred to sterile 50 milliliter (mL) Falcon tubes. Grab samples of the top layers of soil and settled ash were also collected at the base of sugarcane at the sampling location and stored in 50 mL Falcon tubes.

### 2.2. Sample Analysis

#### 2.2.1. Single Particle Analysis

Scanning electron microscopy (SEM) was performed using a field emission system (JEOL model JSM-6500F, Peabody, MA, USA) coupled with energy dispersive spectroscopy (EDS) as previously described [47]. Briefly, a 10-mm section was excised from the parent filter using a hollow steel arch punch, and then sputter-coated with a thin layer of carbon and mounted onto a JEOL microscopy stub. Identification and relative abundances of elements were determined using point and shoot and spectral imaging acquisition modes.

#### 2.2.2. Silica Analysis

X-ray diffraction (XRD) was used to analyze a subset of personal (*n* = 2) and area-based (*n* = 2) PM samples to identify and quantitate the relative contribution of crystalline silica to the PM exposures. A personal cyclone and Button sample containing 120 and 350 micrograms (μg) of PM_2.5_ and PM_100_ were selected. Area-based cyclone and Button samples contained approximately 30 μg each. These samples were sent to an American Industrial Hygiene Association-accredited laboratory that conducted the analysis using a Bruker Endeavor X-ray Diffraction system equipped with a Lynxeye XE-T silicon strip energy dispersive detector in accordance with the Occupational Safety and Health Administration’s Sampling and Analytical Method ID142 [48]. Briefly, filters were dissolved in tetrahydrofuran and redeposited onto a silver membrane using suction filtration. Analytical methods were calibrated and optimized to determine the presence of quartz and cristobalite signal peaks within the sample matrix based on diffraction angles. The reporting levels (RL) are: quartz RL = 10 µg/sample, cristobalite RL = 20 µg/sample and tridymite RL = 20 µg/sample [48].

#### 2.2.3. Total Silica

A new subset of air (*n* = 2) and soil and ash (*n* = 3) samples were analyzed using a Perkin Elmer Elan DRCII Inductively Coupled Plasma Mass Spectrometer (ICP-MS) to obtain the percent of silicon dioxide (silica; SiO_2_) present in the samples as previously described [49]. This analysis does not differentiate between polymorphs of silica (i.e., amorphous and crystalline), but rather it provides a quantitative measure of the total amount of silica present. Briefly, samples were digested in hydrofluoric acid (HF) overnight at 150 °C until the HF acid evaporated and had produced a viscous residue. Reagent and solvent blanks were included in each analysis and served as reference and control for background contamination. Data were reported as a percent mass. 

#### 2.2.4. Heavy Metals Analysis

Given differences in filter digestion methods above, a new subset of samples, which included a 10 mm punch from a personal PM_2.5_ sample and PM_100_ sample, was analyzed using the Perkin Elmer Elan DRCII ICP-MS as described above. Heavy metal concentrations (i.e., arsenic (As), lead (Pb), nickel (Ni), uranium (U), cadmium (Cd), aluminum (Al), barium (Ba), copper (Cu), iron (Fe), indium (In), Sulphur (S), antimony (Sb), titanium (Ti), vanadium (V), and tungsten (W)) were measured in triplicate following extraction and acidification using nitric acid using standard methods [49]. Reagent and solvent blanks were included in each analysis and served as reference and control for background contamination. Data were reported as parts per billion. 

#### 2.2.5. Glyphosate Analysis

A subset of samples were extracted in 1 molar (M) NaOH and mechanically agitated (i.e., vortexing and shaking). The concentration of glyphosate in air and soil extracts was determined using an enzyme-linked immunosorbent assay (ELISA) method (Abraxis LLC; Warmister, PA, USA) according to manufacturer’s instructions. Using a Biotek Synergy™ multi-mode microplate reader at 450 nm, glyphosate concentrations were quantitated using an eight-point standard curve. Sample and assay reagent blanks were included as reference and control to ensure glyphosate-free status of the reagent water, centrifuge tubes, pipette tips, and plates. Positive product controls (i.e., a glyphosate spike) were included to monitor inhibition or enhancement of glyphosate recovery.

## 3. Results

A total of 12 personal air samples were collected (i.e., 6 PM_2.5_ and 6 PM_100_)_._ However, due to a pump failure, only five PM_2.5_ samples were included for analysis. Of those 11 personal air samples, the median sampling time for Sampling Campaign 1 and 2 was 410 min (range: 129–438 min) and 59 min (range: 31–60 min), respectively. Two area-based samples per size fraction were also collected in Sampling Campaign 1 (median sampling time: 468 min) and Sampling Campaign 2 (median sampling time: 62 min). Sampling campaigns were scheduled to incorporate the pre-harvest burn. However, the pre-harvest burn occurred the day before in Sampling Campaign 1 and near the end of shift in Sampling Campaign 2. Hence, sampling times varied between these two campaigns. 

**Figure 2:**
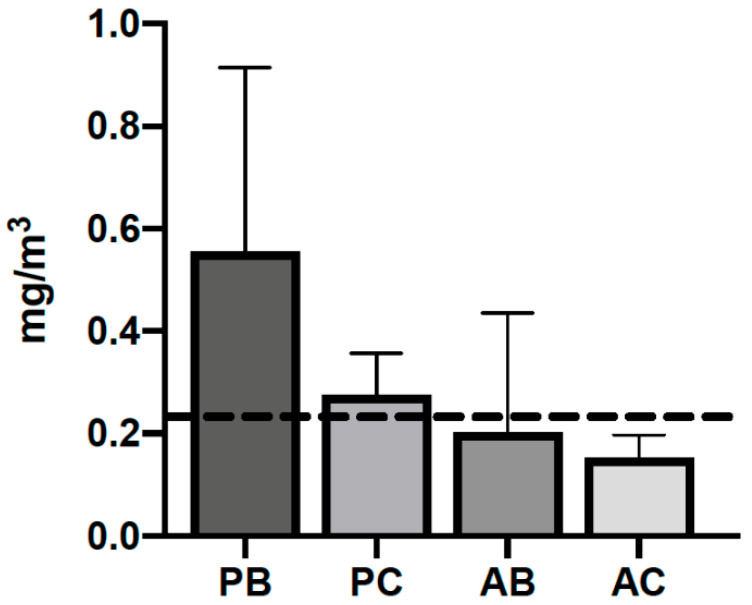
Average mass concentrations by gravimetry across personal Button (PB), personal cyclone (PC), area-based Button (AB), and area-based cyclone (AC) samples with an exposure limit for respirable PM (0.230 mg/m^3^) recommended for agricultural workers.

Personal and area air sampling results are presented in Figure 2. The time-weighted average mass concentration for PM_2.5_ and PM_100_ exposures among worker proxies were 360 µg/m^3^ (range: 32 to 1500 µg/m^3^) and 555 µg/m^3^ (range: 229 to 1170 µg/m^3^), respectively. Despite similar sampling volumes, fixed-site area measurements for PM concentrations were lower than those observed in the personal air samples. For the area measurements, the time-weighted average for PM_2.5_ mass concentration was approximately 90 µg/m^3^ (range: 25–184 µg/m^3^) while PM_100_ concentrations was 203 µg/m^3^ (0.032–536 µg/m^3^).

### 3.1. Silica Results

**Figure 3:**
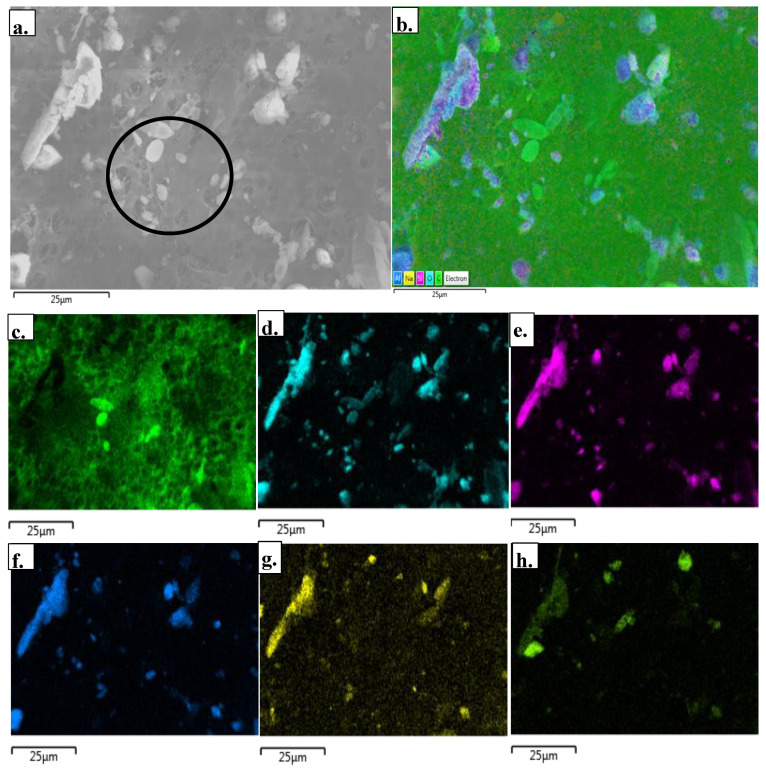
Scanning electron microscopy (SEM) image and energy dispersive spectroscopy (EDS) analysis of a PM_100_ sample. In the specific region of the filter, a wide range of particle sizes and shapes were observed (**a**) including the particulate of biological origin (circle). Elemental maps were generated based on color density to illustrate the relative abundance of elements (**b**), as well as the composition of individual elements (**b**). Overview of all elements present: carbon (**c**), oxygen (**d**), silicon (**e**), aluminum (**f**), sodium (**g**), and calcium (**h**).

**Figure 4:**
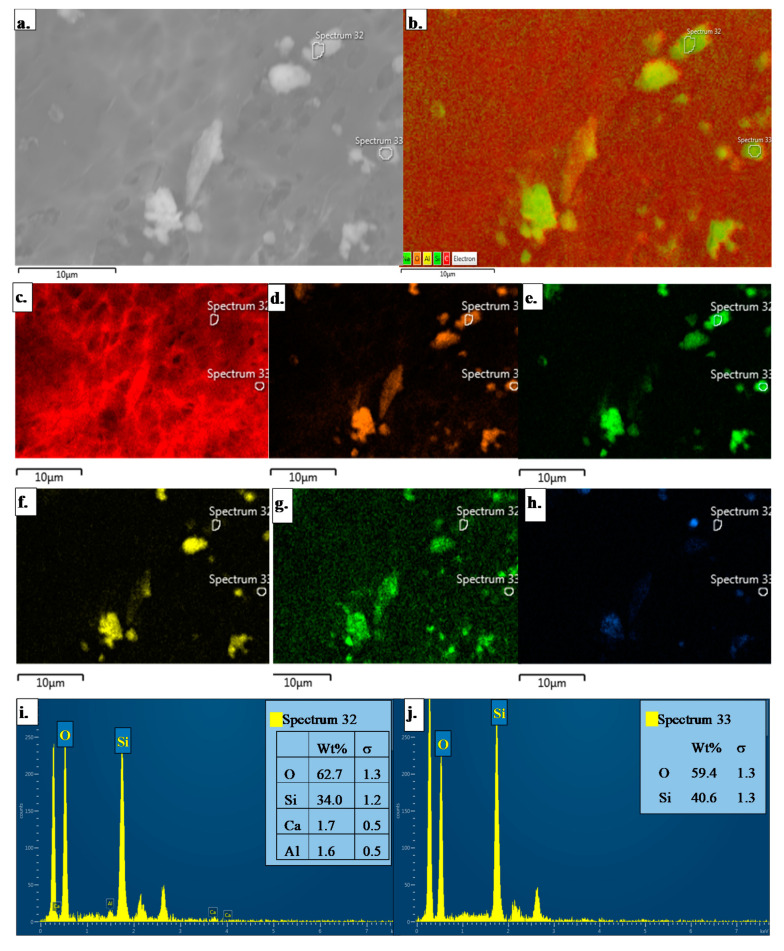
SEM image and EDS analysis of two different single particles present in a personal PM_100_ sample. These particles were <3 µm based on the scale bar (**a**). Based on the elemental map (**b**), these particles did not contain carbon (**c**) and were comprised of only O (**d**) and Si (**e**). No presence of Al (**f**), Na (**g**), and Ca (**h**) were found in these specific particles. Spectrum 32 (**i**) and Spectrum 33 (**j**) further demonstrated that Si and O were most abundant elements and most likely present in the form of silica.

The PM exposures among worker proxies were comprised of particles that varied in morphology and composition based on SEM-EDS (Figure 3a). The elemental composition of particles was driven largely by aluminum (Al), sodium (Na), silicon (Si), oxygen (O), and carbon (C) (Figure 3c–h). However, additional elements such as iron (Fe), magnesium (Mg), and calcium (Ca) were also observed. The carbon signal was largely due to the carbon coating of the sample to minimize contamination and implosion from the electron beam. The signature elements identified were likely in the oxide form, suggesting the presence of feldspar, i.e., aluminum silicates, illite (or phyllosilicate), diopside (volcanic mineral comprised of Mg, Ca, Si, and O), as well as silica in the samples. In a different scan of the sample (Figure 4), a similar signature of elements was observed, including those particles rich with Al and Si. As shown in Figure 4d,e, the spectrum for each particle demonstrated strongly resolved peaks for Si and O. The peak-height of these two elements as compared to other signals (namely background noise), as well as the ratio of the oxygen peak to the silica peak were attributed to the presence of silica. The observed ratio of oxygen to silicon in Spectrum 32 and Spectrum 33 contained in Figure 4i,j was ~1.5 and 2 to 1, respectively. Hence, the ratio of the Si and O energy peaks in these spectra were representative of silica. 

Our results demonstrate that the majority of silica present in these samples is most likely the amorphous form. The amount of quartz (crystalline silica) in the personal (*n* = 2; 1 PM_100_ and 1 PM_2.5_) and area (*n* = 2; 1 PM_100_ and 1 PM_2.5_) air samples analyzed by XRD was below the reporting limit of 5 μg. The percentage of silica measured in the PM_100_ (*n* = 1) and PM_2.5_ (*n* = 1) air samples using ICP-MS were 17% and 18%, respectively (Table 1). Further, the composition of the soil (*n* = 2) sample was 22% and 37% silica by weight while the ash (*n* = 1) sample was just under 60% silica by weight.

### 3.2. Metal Results

The personal air samples were analyzed for other potentially nephrotoxic metals using ICP-MS (*n* = 2; 1 PM_100_ and 1 PM_2.5_). As shown in Table 1, the potentially nephrotoxic metals arsenic, lead, nickel, uranium, and cadmium were not detected. Similar to microscopy results, Al and Ca were present in both size fractions of dust while measurable amounts of gold (Au), In, and Sb were observed. 

### 3.3. Glyphosate Results

No glyphosate was detected in any of the air samples analyzed (*n* = 4). The average concentration of glyphosate present in soil samples (*n* = 4) determined by ELISA was 106 ppb (range: 81–165 ppb; Table 1). The sample collected immediately after a burn operation demonstrated the highest concentration (i.e., 165 ppb). Glyphosate was below the limit of detection in the sample comprised of only settled ash (*n* = 1) (data not presented).

## 4. Discussion

Sugarcane field workers in Guatemala are exposed to potentially high concentrations of PM containing amorphous silica due to emissions from burning and harvesting sugarcane. In this pilot study, exposures to two different size fractions of PM (i.e., PM_2.5_ and PM_100_) were assessed. Our findings suggest that particles exposures are occurring in size fractions that likely impact in the upper airways and that penetrate the unciliated region of the lungs. Concentrations as high as 1500 and 1170 µg/m^3^ were observed in select PM_2.5_ and PM_100_ samples. Of note, silica accounted for almost 20% of the composition by weight in both the personal PM_2.5_ and PM_100_ samples and 60% of the settled ash. Given that the air samplers were worn by research staff (and not actual cane cutters) and sampled for less than full work shifts, these data likely underestimate a worker’s exposure to these hazards. Instead, sugarcane workers are most likely exposed to even higher concentrations of PM and silica based on their continuous activity of cutting and stacking sugarcane, which aerosolizes and/or resuspends PM, ash, and soil into the breathing zone across an entire 8- to 10-h work shift. 

Little is known about the deleterious effects of PM exposure on the kidney. A few recent studies conducted in United States veteran and Medicare populations have found that the concentration of ambient PM_2.5_ is associated with a higher risk of declining kidney function, incident CKD, and end stage renal disease [27,28,29]. For example, in one study, an increased risk for a decline of ≥30% in estimated glomerular filtration rate, eGFR, and end stage renal disease was associated with an increase of 10 µg/m^3^ in the concentration of PM_2.5_. These endpoints were observed even at PM_2.5_ concentrations below thresholds and guidelines promulgated by the World Health Organization (WHO) and the US Environmental Protection Agency (EPA). The WHO and EPA set annual means of 10 and 12 µg/m^3^ for PM_2.5_, respectively, while 24-h means are set at 25 and 35 µg/m^3^.

The average PM_2.5_ concentration observed in this study was 360 µg/m^3^, based on the use of personal air samplers among worker proxies. This concentration is higher than PM_2.5_ concentrations measured during other sugarcane harvest operations in Brazil [21,50]. For example, Santos et al. observed a PM_2.5_ concentration with an interquartile range of 41–87 µg/m^3^ [50] while Leite et al. reported concentrations ranging from 23–33 and 31–139.5 µg/m^3^ during pre-harvest and harvest operations, respectively [21]. Inter-study differences in sampling techniques, duration, and locations were identified, yet no personal air sampling was conducted in the studies conducted by Santos et al. and Letite et al. 

Given the deleterious effects of PM at low levels (described above), exposures at the magnitude observed in our study are a public health concern. Moreover, the exposure assessments in this study were conducted on worker proxies in the field during harvesting activities. As such, exposure limits for occupational populations need to be considered. An occupational exposure limit (OEL) of 230 µg/m^3^ has been recommended for agricultural workers based on the respirable size fraction, which includes those particles with a 50% cut point of 4 µm and lung function [51,52]. While we did not collect respirable samples in this study, it is worth noting that OEL was exceeded despite a smaller size fraction (i.e., PM_2.5_) collected. Moreover, one 8-h sample was ~7× greater than this occupational exposure limit. While the WHO and EPA limits remain important during sugarcane harvest operations, especially for nearby communities, we recognize that the respirable PM is a more physiologically relevant fraction for this occupational population. However, critical questions remain about occupational exposure limits that protect workers against adverse kidney outcomes (i.e., CKDu). Until a causal relation is demonstrated that informs OELs specific to kidney health, assessments are limited to those limits focused on respiratory health 

The health impacts associated with PM_100_ have not been previously considered in sugarcane workers. Those particles ranging from 10–100 µm in aerodynamic diameter are widely recognized to impact in the upper airways [52,53]. Depending on composition and presence of biological and chemical constituents, these larger particles may present unanticipated health risks directly in the nasopharyngeal region or to other parts of the body after clearance from airways and subsequent ingestion [54]. Based on previous research and reports, exposure to PM_100_ plays a key role in the burden of occupational diseases across industrial sectors, including agriculture [55,56,57,58]. As such, we included this size fraction as part of our sampling paradigm. The concentrations of the inhalable fraction observed in this study merit further consideration and investigation to protect the overall health of this vulnerable population.

The silica content observed in the PM samples may play an important role in the etiology of the kidney injury and disease observed in these workers. The soil in southwest Guatemala is rich with silica, likely due to volcanic activity in the area. As a result, appreciable amounts of silica can accumulate in sugarcane plants [59]. The silica present may convert to a crystalline form in the presence of intense heat (>900 °C), which may occur during pre-harvest burning of the sugarcane [60]. The health effects associated with crystalline silica are well documented in other worker populations (e.g., construction) [32,61]. Moreover, CKDu has been reported in miners, construction workers, and brick workers in Central America [1], all of which are work environments associated with silica exposure. Recently, the occupational exposure limit for crystalline silica was reduced to an action level of 25 µg/m^3^ by the Occupational Safety and Health Administration based on silica-related diseases, such as silicosis, lung cancer, and kidney disease [62]. Similar to the results of Le Blond et al., we did not observe levels of crystalline silica above the reporting limit. The silica identified in the PM exposures collected in this pilot study was most likely in the amorphous form. Hence, based on the absence of crystalline silica, we determined that amorphous silica accounted for 17% of the composition by weight. Le Blond et al. demonstrated that residual ash from burning the sugarcane may contain up to 25% amorphous silica by weight and the green sugarcane leaf up to 1.8% by weight [37,63] However, we recognize that we may have collected insufficient amounts of PM to exclude possible exposure to crystalline forms to make this particular determination. Based on our findings and on prior research, both amorphous and crystalline silica should continue to be evaluated. While amorphous silica is thought to be less toxic, its health impacts are not fully understood [64]. Amorphous silica may elicit an inflammatory response that drives kidney injury [65,66].

In this study, we did not observe measurable amounts of arsenic, lead, nickel, uranium, and cadmium in our air samples. However, it is still likely that workers are exposed to these contaminants. For example, a study in 2011 demonstrated that cane cutters (*n* = 45) in Brazil were exposed to airborne cadmium (4.8 ppm) and lead (4.7 ppm) [24]. Notable differences in sampling techniques, analytical methods, study population and sample size between studies may account for the contrasting analytical results. Additional research is needed to ensure collection of representative samples and optimization of sampling and analytical methods to determine if sufficient levels of metals that can initiate kidney injury are inhaled. Further, biomonitoring studies that evaluate urinary metal concentrations in sugarcane workers before, during, and after a harvest operation remain critical to understanding and quantifying real-world exposures. The few Central America studies that have examined the relationship between exposure to nephrotoxic metals and acute or chronic renal effects have yielded varying results [14,67,68,69]. The fingerprint and concentration of heavy metals in the soil have likely changed due to the recent eruption of Fuego in 2018 and subsequent deposition of basaltic ash on the topsoil in the sugarcane fields. This deposition of ash likely contributed varying loads of As, Cd, Ni, Pb, and silica. As such, exposure to nephrotoxic metals among sugarcane workers in Guatemala remains a concern. 

Glyphosate is used routinely in sugarcane fields and has been suggested as a possible contributor to CKDu risk [43]. While glyphosate was not detected in the air samples, it was present in the soil with a maximum concentration of 165 ppb. The lack of glyphosate in our air samples may be due to insufficient PM mass collected, thermal destruction of glyphosate during burning, our inability to extract it from filters, or presence of other substances that might interfere with or inhibit the assay. Since very few studies have reported on airborne concentrations of glyphosate, it remains unclear if this is an important pathway of glyphosate exposure [70,71,72]. Further research is needed to establish optimal collection and measurement methods.

The goal of this study was to generate unique data on exposures to potentially nephrotoxic contaminants present in particulate matter during harvesting of sugarcane in Guatemala. Although our pilot study was limited by small sample size and monitoring worker proxies, our results demonstrate the presence of high PM concentrations containing potentially nephrotoxic contaminants, namely silica. Given that these toxicants are present in PM and plausible pathways for exposure and translocation via the bloodstream to the target organ exist, it is important to fully characterize exposure among sugarcane workers. Thus, further research is needed to completely understand the potential contribution and renal impacts of silica, and potentially heavy metals, via the inhalation pathway. Assessing the association between PM, silica, heavy metals, and glyphosate exposure on sensitive biomarkers of kidney dysfunction will allow researchers to assess whether adverse impacts on the kidney are due to or enhanced by these exposures. There is a particular need to identify mechanisms underlying CKDu progression and to fully characterize air exposures that may be associated with this disease, to inform preventive strategies and improve the health of this vulnerable population. The demonstration of a significant degree of particulate matter emissions stresses the need for reductions in the open burning of sugarcane. Further studies are needed to integrate exposure and health outcomes analyses to assess the health burden attributable to high levels of air pollution in this worker population. 

## 5. Conclusions

The epidemic of CKDu is a major threat to the health of sugarcane and other agricultural workers across the world. To the authors’ knowledge, this pilot study is the first of its kind to investigate the inhalation exposures during sugarcane harvest operations in Guatemala. The data from this pilot study suggests the need to further examine the role of airborne exposures to nephrotoxins and nephrotoxicants, including particulate matter, amorphous silica, metals, and glyphosate. These findings should inform future research including longitudinal study designs that relate air pollutant exposures to kidney health outcomes over time to develop effective control strategies to improve the health of this vulnerable population.

## Figures and Tables

**Table 1:** Concurrent personal air sampling and soil results measured across two days by research staff performing cane cutting.

Inhalation Exposure	Personal PM_2.5_ (*n* = 5) ^a^	Concurrent Personal PM_100_ (*n* = 6)
PM average μg/m^3^ (range)	360 (32–1500)	555 (229–1170)
	**Personal PM_2.5_ (*n* = 1)**	**Concurrent Personal PM_100_ (*n* = 1)**
Total silica ^b^, μg, μg/m^3^, %	9.4, 5.9, 18	28, 115, 17
	**Personal PM_2.5_ (*n* = 1)**	**Concurrent Personal PM_100_ (*n* = 1)**
Crystalline silica ^c^	<LOD	<LOD
	**Personal PM_2.5_ (*n* = 1)**	**Concurrent Personal PM_100_ (*n* = 1)**
Al, Au, Ca, In, Sb, μg/m^3^	6.4, 0.16, 2.0,1.2, 0.41	7.1, 0.08, 6.4, 0.16, 0.0
As, Pb, Ni, U, Cd, μg/m^3^	<LOD	< LOD
	**Personal PM_2.5_ (*n* = 1)**	**Concurrent Personal PM_100_ (*n* = 1)**
Glyphosate average ppb (range)	<LOD	<LOD

^a^ Difference in PM_2.5_ sample size as compared to PM_100_ is attributed to a pump failure (no sample was collected); ^b^ total silica measured via inductively coupled plasma mass spectrometry; ^c^ crystalline silica measured via XRD; not shown: average percent of total silica in soil (*n* = 3) was 38% (range: 22–56%) while the average concentration of glyphosate in soil was 106 ppb (81–165 ppb).
